# Pathological and Epidemiological Characterization of First Outbreak of Daylily Rust in Europe and Evaluation of *Puccinia hemerocallidis* Resistance in *Hemerocallis* Cultivars

**DOI:** 10.3390/plants9040427

**Published:** 2020-03-31

**Authors:** Madalena Ramos, Rita Carvalho, Elsa Soares da Silva, Ana Paula Ramos, Pedro Talhinhas

**Affiliations:** 1 LEAF, Linking Landscape, Environment, Agriculture and Food, Instituto Superior de Agronomia, Universidade de Lisboa, 1349-017 Lisbon, Portugal; madar.mr@hotmail.com (M.R.); silva.elsasoares@gmail.com (E.S.d.S.); pramos@isa.ulisboa.pt (A.P.R.); ptalhinhas@isa.ulisboa.pt (P.T.); 2 LPVVA, Laboratório de Patologia Vegetal “Veríssimo de Almeida”, Instituto Superior de Agronomia, Universidade de Lisboa, 1349-017 Lisbon, Portugal

**Keywords:** quarantine, Portugal, disease, breeding

## Abstract

Daylily rust—caused by *Puccinia hemerocallidis*—was confined to Eastern Asia until the disease was reported in Oceania, Africa, the Americas and Portugal in the 21st century. Although information on rust resistance of American cultivars is available, little is known about the resistance of European bred cultivars, threating the ornamental sector if the fungus spreads to other European countries. Aiming to provide tools to address this, we analyzed the Portuguese pathogens and characterized rust resistance in a selection of cultivars, while optimizing disease rating scales. Morphologic, genetic and cytogenomic characterization of four isolates reveals narrow diversity and raises the question whether the pathogen may have originated in North- or Central America. Daily records of multiple symptomatologic parameters enabled a detailed disease progress analysis, discriminating cultivars according to their resistance levels and revealing susceptibility as the most common state. Among the tested cultivars, 12 out of 17 began to show symptoms between 6–8 dai and were classified as susceptible. Cultivars ‘Stella d’Oro’, ‘Bitsy’ and ‘Cherry Tiger’ behaved as moderately resistant although the occurrence of late sporulation on leaves suggests incomplete resistance and challenges common rating scales. The identification of resistance sources in European breeding lines is crucial for the sustainable future of daylilies.

## 1. Introduction

### 1.1. Daylilies Breeding and Cultivation

**Figure 1:**
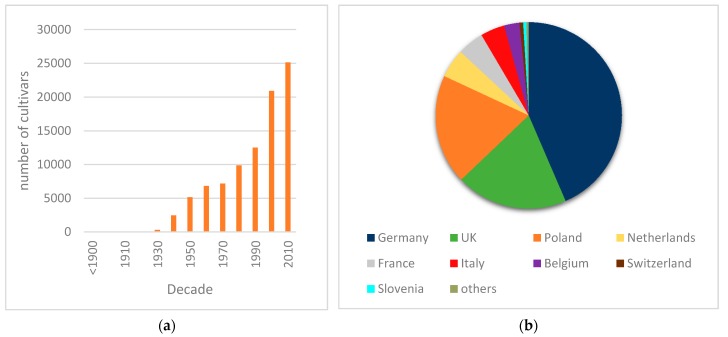
Registered daylily cultivars: (**a**) Number of daylily cultivars registered per decade according to the American Daylily Society database [5]; (**b**) proportion (%) of daylily cultivars registered in Europe per country (total = 2749; ‘others’ include Austria, Belarus and Czech Republic) [7].

Daylilies (*Hemerocallis* spp.; Xanthorrhoeaceae) are originated from Eastern Asia and are widely cultivated as perennial ornamentals from the tropics to their native high latitudes [1]. Besides its ornamental interest, daylily flowers, leaves and roots contain biologically active compounds that have health-enhancing properties [2,3] The genus *Hemerocallis* comprises 20 species, which can be clustered in five groups according to morphologic traits pertaining to the ornamental value and climatic adaptability. Such morphologic diversity, combined with the ease in crossing, led to an intense hybridization by breeders, with an estimated number of 5 million crosses over the last century [4]. The American Daylily Society database [5] lists over 2000 hybridizers and over 90,000 cultivars, a value that has been growing every decade (Figure 1a). Among these, there are over 2500 cultivars of European origin obtained by over 100 hybridizers, most of which active in the last couple of decades, particularly in Germany, Poland, the Netherlands, Belgium, France and Italy (Figure 1b). In most cases daylily breeding is carried out by companies devoted solely and enthusiastically to this crop, giving rise to many exhibitions and competitions. Daylily breeding is thus based on hybridization and progeny selection and is driven by market competition, with numerous, mostly small-scale private players. In Europe, over 30 active breeding companies are currently listed by the European daylily society Hemerocallis Europa e.V. [6].

Daylilies are appreciated not only due to their high ornamental value but also by the extraordinary climatic adaptability, the ease of cultivation and the low disease concerns. However, since the beginning of the 21st century, daylily rust became a worldwide problem. This rust, caused by *Puccinia hemerocallidis* von Thümen [8], is originated from Eastern Asia and was only first reported in Oceania, Africa and the Americas in the first decade of the 21st century [9,10,11,12]. Despite of the efforts of European agencies (e.g., [13,14]), the disease became introduced in several locations in Portugal (mainland and Madeira Island), being first detected in 2015 [15].

### 1.2. Daylily Rust Epidemiology

*Puccinia hemerocallidis* is a macrocyclic fungus, with *Hemerocallis* spp. as the uredinial/telial hosts. The disease symptoms are the typical rust symptoms, with leaves (both pages) showing bright orange spots corresponding to uredosporic sori, leading to chlorosis and senescence. Telial sori appear as dark brown pustules in the autumn. The aecial hosts (*Patrinia* spp.) are basically restricted to Eastern Asia, meaning that elsewhere the disease cycle is limited to the uredosporic cycle. In cool climates daylily plants lose their leaves during winter, hampering the urediniosporic cycle since urediniospores quickly lose viability [16]. However, in mild climates, several daylily cultivars retain foliage during winter, enabling a constant availability of host tissues and thus creating conditions for pathogen dissemination and disease progression [10]. The occurrence of daylily rust in several mild-climate locations in Portugal is therefore of concern, both because the urediniosporic cycle is not broken in these conditions, but also because dominant winds may transport spores eastward to other European countries [17]. Prevalence, incidence and severity were all at high levels in the first record of daylily rust in Portugal [15], suggesting that conditions are favorable for disease spread and development.

The very recent spread of daylily rust from Asia to the other continents means that there is no single study comparing pathogens from all areas where the disease is now present. Hernández et al. [18] have shown that North American isolates have smaller, sparser and less dark telia than Asian specimens and that teliospores are mostly non-septate in the North American fungi, contrasting with mostly two-cell teliospores in Asian samples. The analysis of the rDNA-ITS region supports this division [18], although differences are modest and the inclusion of samples from other areas, such as Oceania and Central America, blurs that separation [17]. The analysis of 16 *P. hemerocallidis* isolates collected in Southeastern USA inoculated on 16 *Hemerocallis* cultivars led Buck [19] to suggest the occurrence of pathotypes with distinct virulence levels, hinting that broader scale studies may shed additional light on the possible existence of physiological races.

### 1.3. Daylily Rust Control

While chemical control of the disease is achievable [20,21], the use of rust resistant cultivars is the most economically and environmentally advisable crop protection strategy. Scales for symptom rating that are both scientifically accurate and feasible to employ in large scale horticultural screening are challenging to obtain. Daylily resistance to rust is based on the hypersensitive reaction (HR), leading to total pathogen arrest (complete resistance) or allowing low levels of sporulation (partial resistance) [20,21]. Highly resistant cultivars arrest fungal growth very early in the infection and HR symptoms are not macroscopically visible, while visible necrotic areas occur in intermediate levels of resistance through to moderately susceptible genotypes. No necroses are found in situations of plain susceptibility, where the fungus produces copious amounts of urediniospores. Following the introduction of the disease in North America, 575 modern local cultivars were evaluated for rust susceptibility under field conditions, with 21% of cultivars showing no or little sign of infection [1]. Another study, performed under more controlled conditions, identified 17% resistant cultivars among 85 North American cultivars [22].

In fact, rust resistance became an important trait for the market in the USA [23]. Yet, no information is available regarding rust resistance among European cultivars and little is known on resistance levels of wild germplasm. Moreover, no information has been compiled about the phylogenetic origin of the resistances identified. In fact, rust resistance breeding is still lagging other breeding traits especially in Europe, urging European breeders and nurseries to become conscious of this problem and of the pathological, environmental and agronomic variables that condition it.

With this work we aim to provide information on daylily rust pertaining to European daylily breeders and nurseries. For such, we have characterized the current prevalence of daylily rust in Portugal, the morphologic, genetic and cytogenomic diversity of its pathogens and the evolution of symptoms as the disease progressed in resistant and susceptible cultivars, as well as the rust resistance in a selection of cultivars.

## 2. Results

### 2.1. Prevalence and Dynamics of Daylily Rust in Portugal

**Figure 2:**
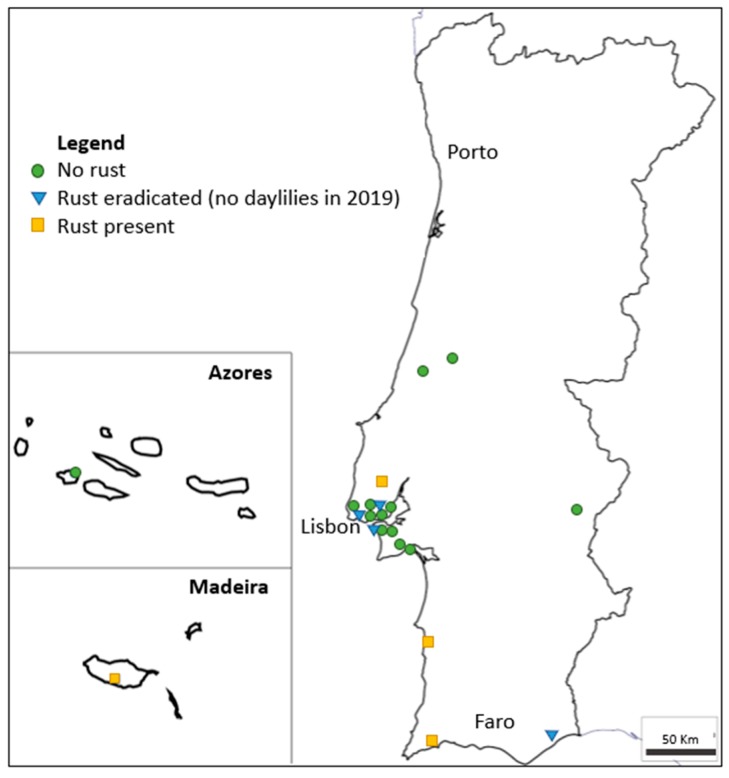
Occurrence of daylily rust (caused by *Puccinia hemerocallidis*) in Portugal according to surveys conducted in 2019, including previous positive detections from which daylily plants (and the disease) were removed (adapted from d-maps.com).

In 2019, twenty-two locations, both public and private gardens, were surveyed throughout the country for daylily rust (Table 1, Figure 2). At fourteen of those locations, the disease was not detected. As in the 2015 survey, in 2019, the disease was present on Madeira Island and in the southeast of Portugal. Two new foci were observed, at Lagos (south of the country) and Sobral de Monte Agraço (near Lisbon), with a high disease incidence and severity record. The national disease prevalence is currently 31%.

### 2.2. Characterization of Puccinia hemerocallidis Populations in Portugal

From the 2019 field surveys, four *P. hemerocallidis* isolates were collected and used for characterization of the pathogen populations present in Portugal (Table 1).

#### 2.2.1. Morphological Characterization

The urediniospores of the four isolates were globose to ellipsoid, pale yellow with a hyaline and uniformly echinulated wall. Only in isolate Ph16 it was observed a pedicel that was attached to some urediniospores. The length and width of the urediniospores of isolates Ph02 and Ph06 had a smaller average size then those of isolates Ph14 and Ph16 (Table 2).

Telia and teliospores were observed only for isolates Ph02 and Ph14. In both cases, teliospores were ellipsoid, brownish, with a pedicel of variable length. The wall of teliospores was smooth and brownish, darkening at the apex. The presence of nonseptate and 1-septate spores was shared by both isolates. Nevertheless, the spores were predominantly nonseptate (87.4%) in isolate Ph14 and slightly less in isolate Ph02 (61.2%). Paraphyses were found only in the Sobral de Monte Agraço isolate.

The average size of the nonseptate and 1-septate teliospores from Porto Covo isolate was not significantly different. On the contrary, in the Sobral de Monte Agraço isolate the only parameter that was not significantly different between the nonseptate and 1-septate spores was the length of the pedicel (Table 3). The average length and width of the two types of teliospores differed between isolates.

#### 2.2.2. Molecular Characterization

**Figure 3:**
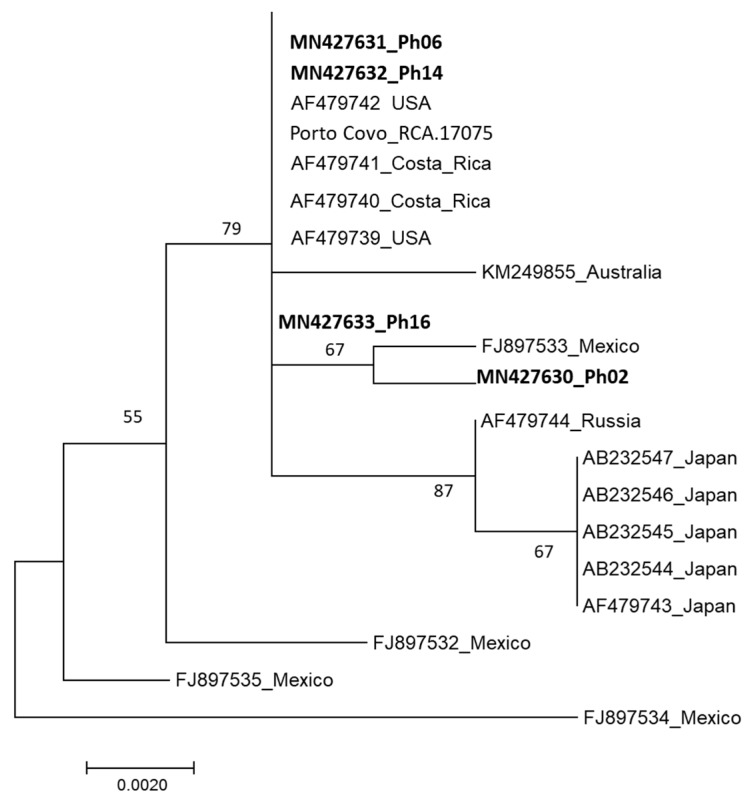
Maximum likelihood (ML) tree generated using rDNA-ITS region for the establishment of phylogenetic relationships between four *Puccinia hemerocallidis* isolates studied and the homologous sequences of isolates from Australia [25], Costa Rica [18], USA [18], Japan [26], Mexico [24], Portugal (Porto Covo; [17]) and Russia [18]. Bootstrap values above 50% are indicated above each branch. The isolates from this study are in **bold** font.

The DNA sequences of the rDNA-ITS region (600 bp) of the four *P. hemerocallidis* isolates were deposited in the GenBank database (Ph02, MN427630; Ph06, MN427631; Ph14, MN427632; Ph16, MN427633). No differences were recorded among isolates except for isolate Ph02, exhibiting differences in two positions. These sequences were compared with homologous sequences of the species from other geographic regions (Figure 3). Isolates Ph06, Ph14 and Ph16 grouped with sequences of isolates from the USA (AF479739, AF479742; [18]) and Costa Rica (AF479740, AF479741; [18]), along with a previous sequence obtained from the pathogen present at Porto Covo location (Porto Covo_RCA17075; [17]). Isolate Ph02 shares a branch in the dendrogram with an isolate from Mexico (FJ897533; [24]).

#### 2.2.3. Cytogenomic Characterization

**Figure 4:**
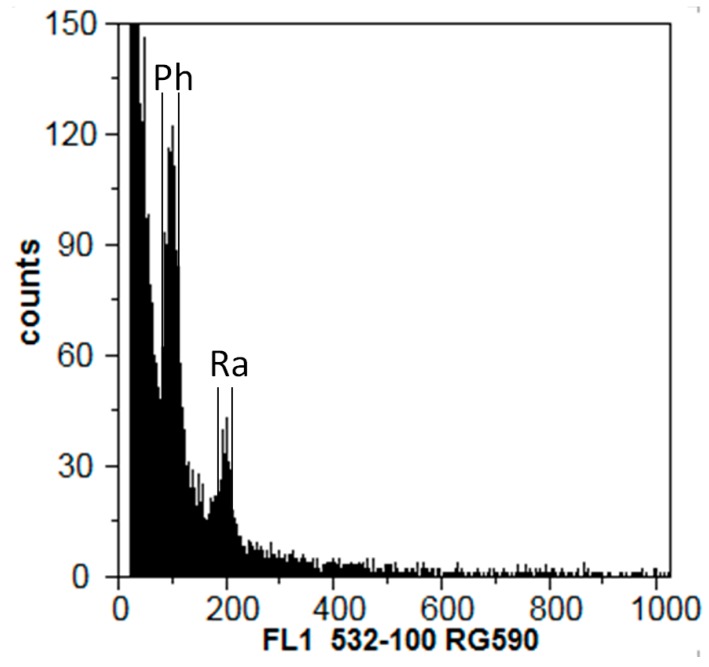
Histogram showing flow cytometric analysis of relative fluorescence intensities (FL1) of propidium iodide-stained nuclei simultaneously isolated from rust-infected daylily plants [*Hemerocallis* sp. (nuclei out of scale) and *Puccinia hemerocallidis* (Ph) isolate Ph02] and the DNA reference *Rhamnus alaternus* (Ra).

Genomic size was estimated by flow cytometry by comparison to *Rhamnus alaternus* L. nuclei (Figure 4). The genomic size of the four isolates ranged from 300 to 325.9 Mbp, showing no significant differences (*p* < 0.05) among isolates (Table 4). A Shapiro–Wilk test (*p* > 0.05) was also performed to check the normality distribution of the data.

### 2.3. Characterization of Daylily Cultivars for Response to Rust

**Figure 5:**
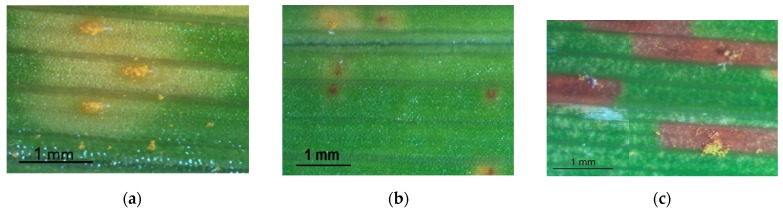
Daylily rust symptoms and signs obtained in susceptible, moderately susceptible and moderately resistant interactions: (**a**) Uredia and urediniospores on the under surface of the leaf in susceptible *Hemerocallis*, observed 7 dai; (**b**) Chlorosis, uredia and small necrotic lesions on the under surface of the leaf in moderately susceptible *Hemerocallis*, observed 16 dai; (**c**) Necrotic lesions on the under surface of the leaf in moderately resistant *Hemerocallis* with urediniospores 30 dai.

The evolution of symptoms varies according to the type of response (Figure 5). Rust susceptible cultivars (Table 5) began to show symptoms 6–8 dai (Table 5). These included the appearance of small chlorotic spots that increased in size during the infection process, leading to yellow streaks (Figure 5a).

**Figure 6:**
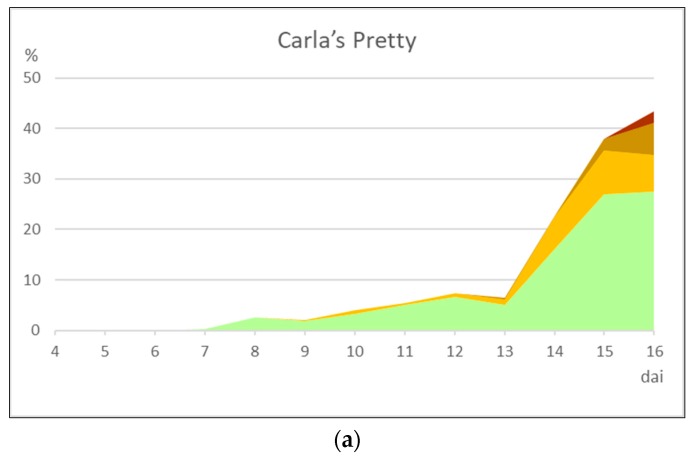

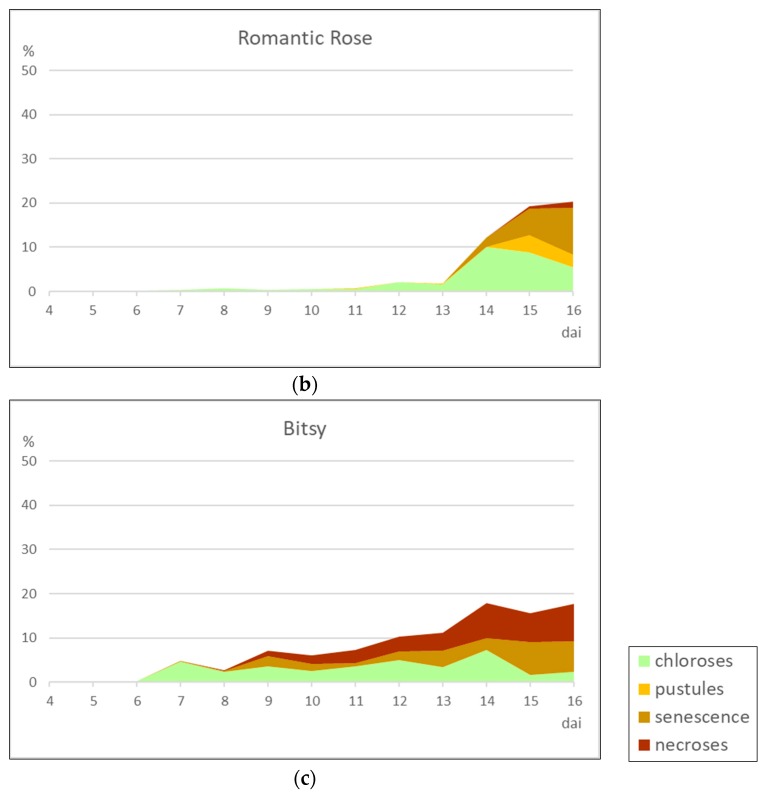
Area under the disease progression curve obtained in susceptible (**a**), moderately susceptible (**b**) and moderately resistant (**c**) interactions between daylilies and *Puccinia hemerocallidis* up to 16 days after inoculation (dai).

The cultivars ‘German Highlight’ and ‘Romantic Rose’ were classified as moderately susceptible due to the appearance of small chlorotic lesions that developed slowly during the trial. On some chloroses there was the development of necrotic lesions, only visible to the magnifying glass (Figure 5b). However, susceptible cultivars presented pustules (Figure 6a) earlier than moderately susceptible cultivars (Figure 6b) and presented more restricted pustule area and diseased area in general.

Moderately resistant cultivars (Figure 6c) developed senescent and necrotic areas soon after displaying chloroses, with necroses becoming prevalent. Sporulation over necrotic areas occurred in these cultivars, but only after the 16-days evaluation period (Figure 5c).

The cultivars that had the longest latent period were the moderately resistant and moderately susceptible cultivars. These also released a smaller number of urediniospores/cm^2^ of leaf, compared to susceptible cultivars that had a smaller latent period (Table 5).

Through the analysis of variance, it was found that there are no significant differences between the upper and under surface of the leaves for all parameters considered (chlorosis, pustules, necrosis and senescence), except for the parameter corresponding to the number of pustules in cultivar ‘Modest Hedwing’, that has the largest number of pustules on the upper surface of the leaves (data not shown).

From the quantification of the diseased leaf area for each cultivar, it was possible to understand that the average percentages recorded for chloroses, pustules, necroses and senescence were not significantly different throughout the trial for the ‘Little Peanut’, ‘Norton Orange’, ‘Norton Signal’, ‘Roswitha’, ‘Rosy Rhino’, ‘German Highlight’ and ‘Romantic Rose’ cultivars. For the moderately resistant cultivars, the total diseased area, including the other symptomatologic parameters, had no significant differences throughout the trial for the ‘Bitsy’ and ‘Cherry Tiger’ cultivars, as opposed to the cultivar ‘Stella d’Oro’, which developed a larger necrotic area.

**Figure 7:**
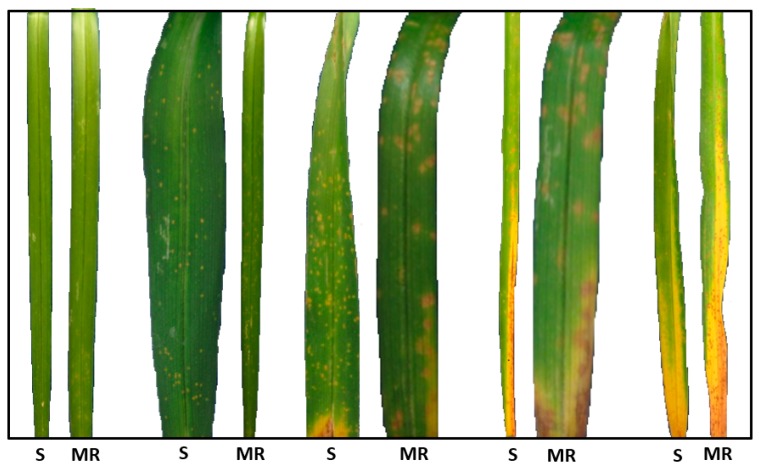
Comparison of diseased leaf area of rust (*Puccinia hemerocallidis*) in leaves of susceptible (S) and moderately resistant (MR) *Hemerocallis* cultivars.

The diseased leaf area of rust in leaves of susceptible and moderately resistant cultivars of *Hemerocallis* was visually compared (Figure 7). The average percentage value of each symptomatologic parameter for the 17 cultivars was statistically analyzed in order to compare the cultivars (Table 6). The cultivars ‘Cherry Tiger’, ‘German Highlight’, ‘Little Peanut’, ‘Norton Orange’, ‘Norton Signal’, ‘Persian Ruby’, ‘Romantic Rose’ and ‘Rosy Rhino’ are not significantly different for the average of area with chloroses, pustules, necroses, senescence and the total diseased area. However, the cultivars ‘Indian Sky’, ‘Roswitha’, ‘Stella d’Oro’ and ‘Watermelon Slice’ have the largest diseased area and are not significantly different. The cultivar ‘Declaration of Love’ stands out, with the largest average area in all parameters, being significantly different from all other cultivars in the values corresponding to the diseased and necrotic area.

An analysis of the genealogy of resistance was performed (based on data available; [27,28]), revealing resistant cultivars that are used as resistance donors, but also identifying uncharacterized cultivars that are potentially resistance sources (Figures S1–S6 and Tables S1 and S2).

## 3. Discussion

Intercontinental spread of plant diseases has played major roles in agriculture and society, among which rusts are of greatest importance, including examples such as wheat stem rust [29], myrtle/eucalyptus rust [30] and coffee leaf rust [31]. Due to their biological properties, rusts are prone to natural and human-driven dissemination, either at short or long distances [32]. In Europe, Portugal (including the foremost position of the Atlantic archipelagos of Azores and Madeira) is placed in a critical position concerning the entry of pathogens (including several rust pathogens) from the Americas or Western Africa, through international trade or natural dissemination [33,34]. After spreading through all continents, daylily rust reached Europe, being detected in several locations in Portugal [15] in 2015. With the goal to prevent further dissemination of the disease in Portugal, some of the initial foci were destroyed. Disease prevalence has thus decreased from 67% in 2015/16 to 31% in 2019, although new occurrences were detected, suggesting some inoculum dissemination or maintenance of routes of introduction of the pathogen. There is no breeding activity of daylilies in Portugal, nor is there any known activity of propagule multiplication, unlike as is the case with several other ornamentals. In countries with cooler winters, such as most European countries, the disease cycle is naturally broken by the loss of daylily foliage, which prevents urediniospore survival. However, climate changes and global warming may contribute to rust dissemination by reducing such wintery periods.

The morphological characteristics of urediniospores and teliospores present in Portugal agreed with the descriptions made by Hernández et al. [18] and von Thümen [8]. The average size of urediniospores among isolates was different. While the urediniospores of isolates Ph02 and Ph06 agreed with the description made by Hernández [18] and von Thümen [8], the average urediniospores size of Ph14 and Ph16 isolates was slightly larger, matching that of urediniospores associated with the first report of *P. hemerocallidis* in South Africa (25.5 × 22 µm; [12]).

The dimensions recorded for the nonseptate and 1-septate teliospores of the Ph02 isolate in accordance with the description made by Thümen [8], Hernández et al. [18], Inokuti et al. [35] and Silva et al. [15]. However, the teliospores of isolate Ph14 had a smaller average size than teliospores found in other geographic regions. The minimum and maximum pedicel length recorded for the two isolates approximate the measurements made by Inokuti et al. [35] and von Thümen [8]. Most teliospores were single celled, similarly to the report by Hernandez et al. [18] for pathogens occurring in North America, contrasting with Asian specimens [18] but also with reports from South America [35,36] where only septate teliospores were found.

The nucleotide sequences of the isolates Ph06, Ph14 and Ph16 did not differ from those of the *P. hemerocallidis* isolates from the USA and Costa Rica. The sequence corresponding to Ph02 isolate was more closely related to the isolate from Mexico. Yet, these differences are based on a very limited number of informative nucleotides. Still, it is worth noting that Asian isolates were clustered together in this analysis, while Mexican isolates were the most diverse. Clearly, a larger set of isolates is needed for this type of study. It is tempting to speculate that more informative markers should be identified and used, although this should be addressed cautiously (the rDNA-ITS region is the universal DNA barcode marker for fungi [37]). One of the objectives of such analyses is to relate genetic diversity to putative physiological races, and this should be considering when choosing appropriate markers. Considering the narrow representativity of the isolates in this analysis and the low levels of diversity revealed, it is still possible to suggest that daylily rust may have entered Portugal in more than one event. Considering morphologic and genetic data, this introduction may have occurred from North or Central America, although the absence of sequences corresponding to isolates from South America, South Africa and Central and Southern Asia does not enable a more thorough analysis.

Considering all measurements made to the size of the *P. hemerocallidis* genome, it is possible to state that the species has a genome with an estimated size of 318 Mbp, matching the single value previously obtained for this species [17], but matching also the average genome size of the order Pucciniales (335 Mbp), one of the largest among fungi [38]. Tavares et al. [39] noted that rust species with uredinial/telial Poaceae hosts have smaller genomes that those infecting Fabaceae. The present genome size value for *P. hemerocallidis* is similar that of *P. allii* (352 Mpb) and of *Uromyces transversalis* (377 Mbp), suggesting that rusts with Asparagales hosts (Xanthorrhoeaceae, Amaryllidaceae and Iridaceae, respectively for those three rust species) have larger genomes that those with Poaceae hosts. Large genomes may be due to activity of transposable elements, chromosome transfer and polyploidization [40], which may be important sources of diversity and variation in genome size, particularly in the absence of sexual reproduction [41]. Genome size analyses of closely related taxa may add information to the events that led to the speciation of *P. hemerocallidis* [26], but they may also be useful for the discrimination of intra-specific diversity of the fungus. To this end, analyses of additional isolates is needed.

In the present study we have analyzed the rust response of 17 daylily cultivars, seven of which of European origin. Three cultivars were classified as moderately resistant, two as moderately susceptible and the remaining as susceptible. Only three of these cultivars had been previously analyzed for rust response. The cultivar ‘Roswitha’ was classified as susceptible to rust in our study, not corresponding to the resistance recorded in previous studies [27]. The cultivar ‘Stella d’Oro’ showed to be moderately resistant, corroborating previous results reporting this cultivar as resistant or moderately resistant [20,21,24,42]. A similar result was found for ‘Bitsy’ [43]. None of the plants analyzed in the present study was considered as resistant, mostly because of late sporulation over necrotic areas, leading to considering them as moderately resistant. Late sporulation, sometimes as late as 30 days after inoculation, may not be recorded in common resistance trials. While immune response is highly desirable as a resistance trait for daylily breeders, other types of hypersensitive response are common in incompatible interactions, often originating macroscopic necroses [21]. Such resistance responses based on macroscopic necroses are less desirable as the ornamental value of the plants depreciate, although the disease cycle is broken. Late sporulation in moderately resistant cultivars does not totally impair inoculum dissemination. Nevertheless, moderate resistance may still be a desirable trait in the absence of higher levels of resistance. Moderately susceptible plants show reduced levels of sporulation and longer latent periods than fully susceptible plants (Table 5) and such quantitative responses may also be used in plant protection schemes to reduce disease while minimizing fungicide application. Disease rating scales in daylily rust must therefore be able to accommodate both qualitative and quantitative variations in disease parameters, as well as to consider the time course of infection. Results from this work, along with those from other authors (e.g., [1,21]), depict necrotic areas as typical of intermediate responses of plants to rust: neither fully susceptible nor fully resistant (immune) responses develop necroses, while moderately susceptible and especially moderately resistant interactions do. A detailed and quantified discrimination of necrotic leaf areas along time, by comparison of healthy areas as well as of diseased (chloroses, pustules and senescence), was shown in this work to be useful to evaluate daylily cultivars. Ideally, disease rating scales should be both scientifically accurate and practical to use in large scale studies. The present image analysis-based study has the potential to be adapted to field surveys using images obtained by robotics or air-driven automated systems.

Among the seven European daylily cultivars analyzed in this study, one was considered as moderately resistant (‘Cherry Tiger’) and two as moderately susceptible (‘German Highlight’ and ‘Romantic Rose’), whereas the other four cultivars shown susceptible to rust. Rust resistance became an important trait for breeders when the disease reached North America, leading to several disease resistance screening studies.

Sources of resistance in European cultivars are however still largely unknown. Interestingly, no clear resistance sources have been reported in wild germplasm, an area that certainly deserves further deepening.

## 4. Materials and Methods

### 4.1. Field Surveys

Daylily plants in public and private gardens were monitored for the occurrence of rust symptoms, including locations previously monitored [15,17]. The prevalence was calculated as the proportion of locations where rust was present. Incidence was calculated as the proportion of diseased plants in each location. Severity was estimated as a proportion of symptomatic leaf area.

### 4.2. Plant Material

*Hemerocallis* sp. plants of an unknown cultivar, previously characterized as susceptible to rust [17], were used for fungal multiplication and production of inoculum. Evaluation of resistance was conducted on 17 daylily cultivars (Table 7) of American or European origins. Plants were kept in 4.3 l pots containing a commercial mixture of universal substrate composed of sieved pine bark humus and peat (Siro Profissional Multiplicação, Leal & Soares S.A., Mira, Portugal) in a greenhouse (transparent 6 mm polycarbonate greenhouse, 27.5 m × 8.5 m) with an average temperature of 24–28 °C and relative humidity of 60%–70%.

### 4.3. Fungal Material

*Puccinia hemerocallidis* isolates were obtained from positive disease detection events during surveys of daylily location. The isolates of daylily rust were collected from infected plant material found at Sobral de Monte Agraço (Ph02), Funchal (Ph06), Porto Covo (Ph14) and Lagos (Ph16) and used for the characterization of pathogen diversity. The characterization of cultivar responses was conducted with the Porto Covo isolate.

### 4.4. Pathogen Characterization

#### 4.4.1. Morphological Characterization of Spores

For microscopy studies, spores were mounted on lactophenol and observed under the microscope at 400× magnification (Leica DM 2500, Wetzlar, Germany). The spores were measured using Leica DFC295 software. To calculate the mean and standard deviation of the length, width and pedicel length the case of teliospores were measured 30 spores for each sample.

#### 4.4.2. Molecular Characterization of rDNA-ITS Region

Daylily leaves infected with each isolate were freeze-dried and subsequently used for genomic DNA extraction since DNA extraction from spores performed unreliably. DNA was extracted using the DNeasy Plant Mini Kit (Qiagen, Hilden, Germany) according to the manufacturer’s instructions and its quality and concentration verified using spectrophotometry and electrophoresis.

The primers ITS1Ext and ITS4Ext [44] were used to amplify the rDNA-ITS region. The reaction mix (10 µL), contained 20 ng DNA, 0.5 μM of each primer and 5 µL of Taq DNA polymerase + dNTPs (NZYTaq II 2× Green) (Nzytech; Lisbon, Portugal). The thermal cycler (UNO II, Biometra, Göttingen, Germany) was programmed as follows: 1 cycle of 5 min at 95 °C, 25 cycles of 30 s at 95 °C, 30 s at 55 °C and 1 min at 72 °C, ending with 1 cycle of 5 min at 72 °C. After amplification and electrophoresis (agarose 1%), the ca. 650 bp bands, corresponding to the fungal rDNA-ITS region [18], were gel-excised and purified with the GeneJET Gel Extraction kit (Thermo Scientific, Waltham, MA, USA), according to the manufacturer’s instructions. DNA extracted from the gel bands was sequenced in each direction with the ITS1Ext and ITS4Ext primers by STABvida (Caparica, Portugal). Sequences were assembled and edited to resolve ambiguities using the EditSeq and Edit Man modules in the Lasergene software (DNASTAR, Madison, WI, USA).

Homologous sequences of *P. hemerocallidis* from other geographic regions were searched using the BLAST algorithm [45] available from NCBI (Table 8). The set of gene sequences were aligned by the ClustalW tool under default parameters, using the MEGA7.014 software and represented by a phylogram constructed by the maximum likelihood method according to the Tamura–Nei model [46]. The confidence levels of the branching points of the tree was determined with bootstrap test, using 1000 bootstrap replicates.

#### 4.4.3. Cytogenomic Characterization of Nuclei

The genome size of each rust isolate was estimated by flow cytometry using a CyFlow Space (Sysmex, Görlitz, Germany), following the procedure of Galbraith et al. [47] adapted by Tavares et al. [39] to rust fungi. To release the rust cell nuclei, infected plant material was chopped with a razor blade along with the internal reference standard *Rhamnus alaternus* L. (2C = 0.680 pg DNA; [17]) in 1 mL Woody Plant Buffer (0.2 mol/l Tris-HCl, 4 mmol/l MgCl_2_, 1% Triton X-100, 2 mmol/l Na_2_EDTA, 86 mmol/l NaCl, 20 mmol/l sodium metabisulfite, 1% PVP-10, pH 7.5; [48]). The nuclear suspension was filtered with a 30 µm nylon filter to remove the debris and 50 µL of propidium iodide (final concentration 50 μg/mL; Sigma-Aldrich, St. Louis, MI, USA) was added to stain the DNA. The suspension within a tube was fed to the flow cytometer and data were acquired using FloMax v2.4d (Sysmex) software. The fluorescence peaks of the *P. hemerocallis* nuclei were identified by comparing the fluorescence histograms of *R. alaternus* leaves. For each isolate five replicates were made.

### 4.5. Characterization of Cultivars for Response to Rust

#### 4.5.1. Inoculation and Incubation

Daylily plants were inoculated following the procedure by Mueller et al. [22] with adaptations. Urediniospores were collected from previously infected plants, suspended in sterile distilled water and sprayed on the leaves at an estimated concentration of 10^6^ urediniospores/mL. The plants were maintained in saturating conditions for 24 h in the dark at 25 °C and thereafter were kept under greenhouse conditions (as mentioned in Section 4.2).

#### 4.5.2. Disease Scoring and Progression

The resistance level of cultivars to rust was evaluated according to the scale described by Li et al. [21] (Table 9). For this, the abaxial and adaxial surface of five leaves of each cultivar were monitored and photographed at 180 ppi with a digital camera (Canon PowerShot A2300) between the 4th and 16th day after inoculation. Images were analyzed according to the instructions in the WinFOLIA (Regent Instruments, Sainte-Foy, Canada) software technical manual. Leaves were chromatically analyzed by decomposing in areas classified as: healthy; chloroses; pustules, necroses; and senescence. The area corresponding to each symptomatic parameter was converted into a percentage according to the total area of each leaf.

Incubation period and latent period were recorded from daily observation of the leaves, using a magnifying glass. Incubation period corresponds to the number of days between inoculation and observation of the first macroscopic symptoms; latent period corresponds to the days between inoculation and release of the first urediniospores from uredia [21]. Urediniospore production was quantified 16 day after inoculation, according to the protocol by Li et al. [21].

### 4.6. Statistical Analysis

Statistical analyses were processed using Statistica 6.0 software (StatSoft, Tulsa, OK, USA). Data regarding the percentage of leaf area affected by chloroses, pustules, necroses, senescence and, consequently the total percentage of diseased leaf area were treated statistically by analysis of variance (ANOVA), as well as biometric variables of urediniospores (length and width) and the genome size of each rust isolate. To test for existence significant differences between variable means was applied Tukey test (*p* < 0.05).

To test the Normal distribution on the data were performed a Shapiro–Wilk test (*p* > 0.05) using the software RStudio (RStudio, Boston, MA, USA).

## 5. Conclusions

After spreading from Asia to all other continents in the 21st century, daylily rust has entered Europe through its Southwestern point. Four years after being detected in multiple locations in mainland Portugal and Madeira, this study showed that although the prevalence of the disease decreased since 2016, new occurrences were identified and showed a high incidence and severity.

Morphologic, genetic and genomic analyses show that the pathogens present in Portugal exhibit narrow diversity, suggesting that the pathogen may have been introduced in Europe from North or Central America. Results obtained in this study indicate a need for a global study comparing *Puccinia hemerocallidis* isolates, mainly aiming at the identification of physiological races.

Detailed analysis of the daily evolution of diverse symptomatological parameters has enabled providing qualitative and quantitative data for accurate classification of cultivars. Additionally, the phenomenon of late sporulation over necrotic tissues was highlighted, suggesting that cultivars classified as resistant may be better characterized as moderately resistant.

Experience from the American continent shows that, once introduced, daylily rust is unlikely to be eradicated. Either naturally or human-driven, daylily rust has potential to reach other parts of Europe and compromise breeding and nursery activities of the ornamental horticulture sector. This study emphasizes the fact that rust response in European cultivars is largely unknown, although resistant germplasm is available and commonly used in breeding programs. Achievement of knowledge of the resistance levels of European daylily cultivars, including also wild genotypes, is required to maintain daylilies as disease-free prime-choice garden plants. It is also urgent that plant breeders, especially those who only breed *Hemerocallis* spp., take awareness of the resistance level of the existing varieties to avoid future economic losses.

## Figures and Tables

**Table 1:** Survey of daylily rust ^1^ in Portugal in 2019, compared to previous surveys (in 2015 or 2016; [15]), including the reference of the isolate and the evolution of national prevalence.

Location	2015/16	2019
Oeiras	1	n
Avenidas Novas_1, Lisbon	1	n
Almada	1	n
Tavira	1	n
Funchal, Madeira	1	1
Porto Covo, Sines	1	1
Sobral de Monte Agraço	-	1
Lagos	-	1
Alcântara, Lisbon	0	0
Ajuda, Lisbon	0	0
Palmela	0	0
Campo Grande, Lisbon	-	0
Santo António, Lisbon	-	0
Avenidas Novas_2, Lisbon	-	0
Sintra	-	0
Corroios, Seixal	-	0
Amora, Seixal	-	0
Setúbal	-	0
Vila Viçosa	-	0
Horta, Faial, Azores	-	0
Maceira, Leiria	-	0
Avelar, Ansião	-	0
Prevalence	67%	31%

^1^ 1, Presence of rust symptoms; 0, absence of rust symptoms; n, no daylily plants present; -, no survey.

**Table 2:** Urediniospores dimensions (in µm) of four *Puccinia hemerocallidis* isolates collected in Portugal.

Isolates	Length (L)		Width (W)		L/W
	Min–Max	Mean	Min–Max	Mean	Mean
Ph02	19.4–27.4	22.8 ± 1.8 a ^1^	17.5–22.2	20.5 ± 1.1 a	1.11 ± 0.1 a
Ph06	19.1–29.5	23.8 ± 2.4 a	17.7–23.6	20.4 ± 1.4 a	1.17 ± 0.1 a
Ph14	20.6–30.9	25.4 ± 2.3 b	19.2–25.5	22.1 ± 1.3 b	1.15 ± 0.1 a
Ph16	21.8–31.0	25.3 ± 2.1 b	19.7–25.2	22.7 ± 1.3 b	1.12 ± 0.1 a

^1^ In each column, the mean values followed by the same letter are not significantly different according to Tukey test (*p* < 0.05). A Shapiro–Wilk test was performed to verify the normal distribution of the values (*p* > 0.05).

**Table 3:** Teliospores dimensions (in µm) of two *Puccinia hemerocallidis* isolates collected in Portugal.

Iso.	Septa		Length (L)		Width (W)		Pedicel		L/W
	No.	%	Min–Max	Mean	Min–Max	Mean	Min–Max	Mean	Mean
Ph02	0	61.2	26.9–47.8	35.9 ± 5.6 a ^1^	11.9–19.3	15.2 ± 1.7 a	7.3–28.0	18.1 ± 6.3 a	2.4 ± 0.4 a
	1	38.7	35.0–55.0	43.0 ± 4.6 b	8.5–18.8	13.3 ± 2.7 b	7.6–28.8	14.5 ± 5.4 ab	3.4 ± 0.9 b
Ph14	0	87.4	16.4–43.8	25.8 ± 8.7 c	5.0–15.7	9.1 ± 3.0 c	5.3–29.2	12.3 ± 6.2 b	2.9 ± 0.6 c
	1	12.6	19.1–45.4	23.8 ± 5.2 c	5.3–14.7	7.8 ± 2.0 c	6.2–25.8	10.7 ± 4.3 b	3.1 ± 0.6 bc

^1^ In each column, the mean values followed by the same letter are not significantly different according to Tukey test (*p* < 0.05). A Shapiro–Wilk test was performed to verify the normal distribution of the values (*p* > 0.05).

**Table 4:** Genome size (in Mbp, and the corresponding coefficient of variation, CV) of four *Puccinia hemerocallidis* isolates.

Isolate	Mean 1C (Mbp)	CV (%)
Ph02	304.0 a ^1^	6.7
Ph06	300.0 a	7.6
Ph14	325.9 a	7.0
Ph16	313.5 a	7.6
average	310.9	7.2

^1^ Mean values followed by the same letter are not significantly different according to Tukey test (*p* < 0.05). A Shapiro–Wilk test (*p* > 0.05) was also performed to check the normality distribution of the data.

**Table 5:** Incubation period, latent period, sporulation and resistance category for 17 *Hemerocallis* cultivars inoculated with *Puccinia hemerocallis*.

Cultivar ^1^	Incubation Period (Days)	Latent Period (Days)	Sporulation (×10^3^ Urediniospores/cm^2^ of Leaf Area 16 dai) ^2^	Resistance Category ^3^
Jerry Nettles	-	-	-	S
Raspberries in cream	-	-	-	S
Wild Cherry	-	-	-	S
Carla’s Pretty	7	9	96.5	S
Declaration of Love	6	9	18.8	S
Norton Orange	4	10	210	S
Norton Signal	4	10	44.0	S
Indian Sky	6	12	21.7	S
Modest Hedwing	7	12	102	S
Persian Ruby	7	12	31.9	S
Stellar Double Rose	6	12	40.3	S
Watermelon Slice	6	12	73.5	S
Roswitha	5	13	170	S
Rosy Rhino	6	13	15.0	S
Little Peanut	6	14	93.3	S
German Highlight	4	14	67.2	MS
Romantic Rose	6	14	13.6	MS
Stella d’Oro	6	12	13.6	MR
Bitsy	6	>16	4.47	MR
Cherry Tiger	6	>16	3.57	MR

^1.^ The cultivars ‘Jerry Nettle’, ‘Raspberries in cream’ and ‘Wild Cherry’ showed susceptibility to rust under field conditions in the *Hemerocallis* population identified at Sobral de Monte Agraço. ^2.^ Dai—Days after inoculation. ^3.^ MR—Moderately resistant; MS—Moderately susceptible; S—Susceptible.

**Table 6:** Average percentage of leaf area corresponding to chloroses, pustules, necrosis, senescence and total diseased area for each cultivar of *Hemerocallis* inoculated with *Puccinia hemerocallidis* and respective statistical analysis using the Tukey test (*p* < 0.05).

Cultivar	Resistance Category ^2^	Chloroses	Pustules	Necroses	Senescence	Total Diseased Area
German Highlight	MS	2.14 a ^1^	0.01 a	0.00 a	0.00 ab	2.16 ab
Norton Orange	S	1.99 a	0.27 a	0.00 a	0.01 a	2.27 a
Norton Signal	S	2.06 a	0.34 a	0.00 a	0.00 a	2.40 a
Persian Ruby	S	1.83 a	0.41 ab	0.00 a	0.28 ab	2.52 ab
Romantic Rose	MS	2.36 a	0.54 ab	0.14 a	1.45 ab	4.49 abc
Little Peanut	S	3.07 a	1.30 abc	0.21 a	0.49 ab	5.07 abc
Cherry Tiger	MR	5.80 abc	0.00 a	0.07 a	0.08 a	5.95 abcd
Rosy Rhino	S	5.38 ab	0.88 ab	0.54 a	0.04 a	6.84 abcd
Bitsy	MR	2.81 a	0.00 a	2.85 ab	2.08 ab	7.74 abcd
Modest Hedwing	S	6.05 abcd	2.34 abcd	0.00 a	0.34 ab	8.73 abcde
Carla’s Pretty	S	7.34 bcde	1.93 abc	0.19 a	0.71 ab	10.16 bcde
Stellar Double Rose	S	5.88 abc	3.41 abcd	1.07 ab	0.00 a	10.36 cde
Roswitha	S	9.91 de	1.29 abc	0.26 a	0.34 a	11.80 def
Watermelon Slice	S	10.30 e	0.80 abcd	0.59 a	3.18 abc	14.87 ef
Stella d’Oro	MR	9.29 cde	0.00 a	6.23 abc	0.14 a	15.67 ef
Indian Sky	S	4.52 ab	2.77 abcd	4.94 abc	5.77 abcd	18.00 f
Declaration of Love	S	9.27 bcde	3.12 abcd	12.72 d	8.04 abcd	33.15 g

^1.^ Mean values followed by the same letter are not significantly different according to Tukey test (*p* < 0.05). ^2.^ MR: Moderately resistant; MS: Moderately susceptible; S: Susceptible.

**Table 7:** *Hemerocallis* cultivars used in this study, bloom season, winter foliage, ploidy, year of registration and parentage.

Cultivar	BS ^1^	WF ^2^	P ^3^	C ^4^	Year ^5^	Originator ^6^	Parentage ^7^
Bitsy	EE	S	D	USA	1963	Warner	Pinocchio × Sooner Gold
Carla’s Pretty	MLa	S	T	NL	2006	Heemskerk	-^8^
Cherry Tiger	M	S	T	NL	2006	Heemskerk	-
Declaration of Love	EM	D	T	DE	1998	Reinermann	Tigger × German Transrapid
German Highlight	EM	D	D	DE	1994	Reinermann	Rudolf Seyer × Siloam Shocker
Indian Sky	M	E	D	USA	1963	Farris	-
Little Peanut	EM	D	D	USA	1985	Winniford-E.	Dallas Lass × Poe’s Raven
Modest Hedwing	-	-	T	USA	-	-	-
Norton Orange	EM	E	T	UK	1971	Coe	-
Norton Signal	MLa	D	T	UK	1971	Coe	-
Persian Ruby	EM	D	T	USA	1998	Trimmer	Ruby Spider × (Nordic Night × Tet Regal Finale)
Romantic Rose	EM	D	D	NL	2013	Heemskerk	-
Roswitha	EM	D	D	USA	1992	Trimmer	Exotic Echo × Janice Brown
Rosy Rhino	M	S	T	USA	2001	Salter	-
Stella d’Oro	EM	D	D	USA	1975	Jablonski	-
Stellar Double Rose	EM	D	D	USA	1995	Brown-C.	-
Watermelon Slice	E	E	T	USA	1998	Scott-E.	-

^1^ Bloom season: EE—Extra early; E—Early; EM—Early midseason; M—Midseason; MLa—Late Midseason [5]; ^2^ Winter foliage: D—Dormant; E—Evergreen; S—Semievergreen [5]; ^3^ Ploidy: D—Diploid; T—Tetraploid [5]; ^4^ Country: NL—Netherlands; DE—Germany; UK—United Kingdom [5,7]; ^5^ Year of registration with the American Hemerocallis Society [5]; ^6^ Originator of the cultivar [5]; ^7^ Pod parent × Pollen parent; ^8^ unknown.

**Table 8:** Host, isolate number, country and GenBank accession of *Puccinia hemerocallidis* included in the phylogenetic analyses.

Isolate No.	Country	Host	GenBank Accession	Reference
Ph02	Portugal	*Hemerocallis* sp. cv. Wild Cherry	MN427630	this study
Ph06	Portugal	*Hemerocallis* sp.	MN427631	this study
Ph14	Portugal	*Hemerocallis* sp.	MN427632	this study
Ph16	Portugal	*Hemerocallis* sp.	MN427633	this study
Porto Covo RCA17075	Portugal	*Hemerocallis* sp.	-	[17]
BPI746996	USA	*Hemerocallis* sp.	AF479739	[18]
BPI748483	Costa Rica	*Hemerocallis* sp.	AF479740	[18]
BPI749105	Costa Rica	*Hemerocallis* sp. cv. Little Dandy	AF479741	[18]
BPI841369	USA	*Hemerocallis* sp.	AF479742	[18]
BPI840988	USA	*Hemerocallis* sp.	AF479744	[18]
Chicol 2	Mexico	*Hemerocallis* sp.	FJ897533	[24]
Chicol 1	Mexico	*Hemerocallis* sp.	FJ897532	[24]
Verac 3	Mexico	*Hemerocallis* sp.	FJ897534	[24]
Verac 4	Mexico	*Hemerocallis* sp.	FJ897535	[24]
BRIP:53476	Australia	*Hemerocallis* sp.	KM249855	[25]
IBA8568	Japan	*Patrinia villosa* (Thunb.) Juss.	AB232543	[26]
IBA8745	Japan	*Hemerocallis fulva* L. var. longituba (Miq.) Maxim.	AB232544	[26]
AB232546	Japan	*Hemerocallis fulva* L. var. longituba (Miq.) Maxim.	AB232545	[26]
IBA8749	Japan	*Hemerocallis fulva* L. var. longituba (Miq.) Maxim.	AB232546	[26]
IBA8759	Japan	*Hemerocallis fulva* L. var. longituba (Miq.) Maxim.	AB232547	[26]

**Table 9:** Categories of resistance of daylilies (*Hemerocallis* spp.) to rust (*Puccinia hemerocallidis*) according to (and adapted from) Li et al. [21].

Category	Description
Susceptible	Yellow lesions; abundant sporulation, no necroses
Moderately susceptible	Sporulation with some necrotic lesions
Moderately resistant	Abundant necrotic lesions with a few sporulating infections
Resistant	Necrotic lesions, no sporulation
Highly resistant	No macroscopically visible symptoms

## Supplementary Materials

The following are available online at https://www.mdpi.com/2223-7747/9/4/427/s1, Figure S1: Genealogy of rust resistance related to daylily cultivars Lullaby Baby and Ming Porcelain. Figure S2: Genealogy of rust resistance related to daylily cultivar Ruffled Ivory. Figure S3: Genealogy of rust resistance related to daylily cultivars Super Fancy Face and Texas Kaleidoscope. Figure S4: Genealogy of rust resistance related to daylily cultivar Super Purple. Figure S5: Genealogy of rust resistance related to daylily cultivars Fairy Tale Pink, Neal Berrey and Ronda. Figure S6: Genealogy of rust resistance related to daylily cultivars Little Infant, Preppy and Joan Senior., Table S1: Main traits and genealogy of daylily cultivars (and progenitors) showing resistance to rust. Table S2: Main traits and genealogy of daylily cultivars having rust-resistant cultivars in their genealogy. Source: the American Daylily Society database [3].
